# Impact of a maternal and newborn health results-based financing intervention (RBF4MNH) on stillbirth: a cross-sectional comparison in four districts in Malawi

**DOI:** 10.1186/s12884-021-03867-6

**Published:** 2021-06-05

**Authors:** Regina Makuluni, William Stones

**Affiliations:** 1 College of Medicine, The University of Malawi, Private Bag 360, Blantyre, Malawi South Africa; 2 Center for Reproductive health, College of Medicine, The University of Malawi, Private Bag 360, Blantyre, Malawi South Africa

## Abstract

**Background:**

Malawi implemented a Results Based Financing (RBF) model for Maternal and Newborn Health, “RBF4MNH” at public hospitals in four Districts, with the aim of improving health outcomes. We used this context to seek evidence for the impact of this intervention on rates of antepartum and intrapartum stillbirth, taking women’s risk factors into account.

**Methods:**

We used maternity unit delivery registers at hospitals in four districts of Malawi to obtain information about stillbirths. We purposively selected two districts hosting the RBF4MNH intervention and two non-intervention districts for comparison. Data were extracted from the maternity registers and used to develop logistic regression models for variables associated with fresh and macerated stillbirth.

**Results:**

We identified 67 stillbirths among 2772 deliveries representing 24.1 per 1000 live births of which 52% (*n* = 35) were fresh (intrapartum) stillbirths and 48% (*n* = 32) were macerated (antepartum) losses. Adjusted odds ratios (aOR) for fresh and macerated stillbirth at RBF versus non-RBF sites were 2.67 (95%CI 1.24 to 5.57, *P* = 0.01) and 7.27 (95%CI 2.74 to 19.25 *P* < 0.001) respectively. Among the risk factors examined, gestational age at delivery was significantly associated with increased odds of stillbirth.

**Conclusion:**

The study did not identify a positive impact of this RBF model on the risk of fresh or macerated stillbirth. Within the scientific limitations of this non-randomised study using routinely collected health service data, the findings point to a need for rigorously designed and tested interventions to strengthen service delivery with a focus on the elements needed to ensure quality of intrapartum care, in order to reduce the burden of stillbirths.

## Background

The 2015 global estimate for stillbirths was 18.4 per 1000 live births, a 25.5% reduction from 24.7 in 2000 [1]. Expressed as absolute numbers this corresponded in 2015 to 2.6 million losses, representing a 19% decline since 2000. However, the smallest reduction was documented in sub-Saharan Africa [1]. Until recently, deaths of fetuses before and during labour have remained relatively invisible to policymakers, development partners and funding agencies, despite the commonalities between risk factors for stillbirth and those for neonatal mortality [2]. A global target for stillbirth has been set at 12 per 1000 live births by 2030 against the current 18.4 per 1000 live births [3]. Across all settings, poor quality health care is an important risk factor for stillbirth. Both lack of access to antenatal care and suboptimal quality of intrapartum obstetric care have been highlighted as increasing the risk of stillbirth [4].

While improved access to and quality of antenatal care may prevent instances of antepartum fetal loss through detection of conditions such as pre-eclampsia, improved obstetric care during labour and delivery has a major role to play in prevention of intrapartum (fresh) stillbirth [5]. Just under half of all stillbirths in low and middle income countries occur during labour and delivery [6]. By contrast, in high income countries these deaths largely occur before labour and the intrapartum fetal death of an apparently healthy fetus has become rare, suggesting that with optimal obstetric care, most of these stillbirths are preventable [4, 6]. In Malawi, the overall stillbirth rate is estimated at 24 per 1000 births [3, 7].

As a programmatic response, the Government of Malawi through the Ministry of Health Reproductive Health Directorate (RHD) adopted an initiative in 2013 entitled “Results-Based Financing for Maternal and Newborn Health” (RBF4MNH) in four Districts, Dedza, Ntcheu, Mchinji and Balaka. The aim of the initiative was to improve the quality of maternal and newborn care services while maintaining high rates of service utilization in both public and selected private not-for-profit facilities [8]. The initiative included both supply and demand-side incentives with a supply-side intervention (Performance Based Financing, PBF) that comprised financial rewards to health facilities attaining pre-defined indicators of clinical and organizational performance in labour, delivery, and newborn care [9]. The financial allocation was to be utilised depending on the hospital’s needs for investment in promoting quality maternal and newborn health care. A demand-side component was to facilitate access and was provided through cash transfers to pregnant women.

The programme had a strong focus on increased quality of maternal and neonatal health care, staff motivation, providing monetary support to pregnant women to reduce access gaps and extending community awareness of institutional deliveries. Stillbirth rate reduction was not specified as a performance indicator. The goal of RBF was to enable more women, particularly from poor rural areas, to deliver in health facilities, and for those facilities to offer better quality maternal and neonatal care services [10]. Typically, in Malawi a woman stays in hospital for a maximum of 24 h after normal delivery owing to space constraints while in RBF hospitals a stay of 48 h post-delivery was mandated [11]. Besides performance contracts, the RBF4MNH initiative also included an initial input component to address basic infrastructural needs and addressing of minor building repairs [10]. Separate sets of performance incentives were set up for the District Health Management Teams (DHMT) in each of the four districts.

Apart from receiving incentives, health workers underwent training and received mentorship in the provision of care to women and newborns. The intervention’s impact on maternal and newborn health service utilization and quality of care has been reported [12]. Here, we undertook a comparison of intervention and non-intervention sites to assess the impact on stillbirth.

## Methods

This was a quantitative cross-sectional study which used routinely collected hospital data in the referral hospitals serving the districts of Ntcheu, Dedza, Salima (Central region) and Thyolo (Southern region). These district hospitals provide a secondary level of care and serve as the referral hospitals for all the primary health centres in their respective districts. The RBF districts were purposively selected because of ready access to hospital records. The non-RBF comparison districts were randomly selected by applying a random number table to a list of Malawi districts. The primary outcome for analysis was stillbirth, defined as an infant born with no signs of life at or after 28 weeks gestation [13].

A power calculation to determine the sufficiency of the number of register records was performed using Open Epi version 3 resulting in a sample of 2800 with a power of 90%, at a statistical significance level of 5%. This was based on anticipated stillbirth rates of 15 per 1000 live births in the combined population of the intervention hospitals and 34 per 1000 in the combined population in the non-intervention hospitals. These assumptions were based on initial scrutiny of District Health Management Information System (DHIS-2) returns.

Data were extracted from the registers which are used in maternity units to prepare routine monthly reports. Data collected included maternal age, gravidity (number of pregnancies), and gestational age in weeks, preeclampsia, low birth weight and stillbirths. We used assessment of gestational age and birth weight recorded in the registers following the routine practice of health facilities. In Malawi, most women do not undergo sonography to confirm gestational age. In these hospitals, weighing of the newborns is done by the midwives using routinely available hospital weighing scales. A prepared data extraction tool in the form of a register was used. Data were entered into Microsoft Excel and checked for accuracy, consistency, and completeness. Analysis was undertaken using STATA version 14. For continuous variables, means and standard deviations were considered and presented. Logistic regression models were developed to determine the odds ratio for stillbirth under intervention and non-intervention conditions, and to assess whether the statistical relationship was confounded by other factors. The possible confounders included in the multivariable models were; low birth weight, gestational age, gravidity and a diagnosis of pre-eclampsia, based on findings in two previous local studies [6, 7].

The study was approved on a waiver of the need for individual participant consent by the nationally mandated College of Medicine Research and Ethics Committee (COMREC) and administrative permission for access was granted by the Hospital authorities.

## Results

We obtained records of 2800 births, of which 28 (1%) were discarded during data cleaning owing to incorrect and missing values. Of these, 0.6% (*n* = 8) observations were dropped at RBF sites while 1.4% (*n* = 20) observations were dropped in the non-RBF sites. A total of 2772 delivery records were used in the final analyses. One thousand three hundred ninety-two of the deliveries were from RBF sites representing 50.2% while 1380 were from non-RBF sites representing 49.8%.

### Demographic characteristics of the mothers with stillbirths

The mean maternal age was 24 years (SD: 6.66). Most of the women (43.3%) were aged between 25 and 34 with 4.5% aged below 18 years. Just under half (47.8%, *n* = 32) were in their first or second pregnancy as shown in Table 1 below.

**Table 1:** Demographic characteristics of the mothers with stillbirths

Variable		Frequency	Percentage
Age (years)	Less than 18	3	4.5
	18 to 19	11	16.4
	20 to 24	18	26.9
	25 to 34	29	43.3
	35+	6	8.9
Number of pregnancies	1 to 2	32	47.8
	3+	35	52.2
Gestational age (Weeks)	28 to 33	12	17.9
	34to 36	10	14.9
	37 to 39	41	61.3
	40 to 42	4	5.9
	43 above	0	0

### Factors associated with stillbirth

During the study period, 67 stillbirths were identified out of 2772 deliveries. Fifty-two percent (*n* = 35) were classified as fresh (intrapartum) stillbirths while 48% (*n* = 32) were macerated (antepartum) stillbirths in the maternity unit registers. Regarding gestational age of the entire sample population, 16.7% (*n* = 463) were preterm and 83.3% (*n* = 2309) presented at term.

There were 1340 live births at the RBF sites and 1365 live births at non-RBF sites representing 96.3 and 98.9% of all births respectively (Table 2). More stillbirths were observed in the RBF sites than non-RBF sites, 3.8 and 1.1% respectively (*P* < 0.001). Other bivariate associations noted were with gestational age: of the 441 preterm births, 22 (4.7%) were stillbirths (*P* < 0.001) and low birth weight: of the 263 newborns with low birth weight, 4.7% were stillbirths (*P* = 0.01). Other potential associations were non-significant.

**Table 2:** Factors associated with stillbirth, comparison between RBF and non-RBF sites

Variable	Frequency	Live (%)	Stillbirth (%)	*P* Value
RBF				
Yes	52	96.3	3.7	< 0.001
No	15	98.9	1.1	
Age group				
Below 18	3	98.8	1.2	0.108
18–19	11	97.9	2.1	
20–24	18	97.9	2.1	
25–34	29	96.4	3.6	
35+	6	98.2	1.8	
Gravidity				0.256
Primigravida	23	97.9	2.1	
2–5	36	97.5	2.5	
6+	8	96.6	3.4	
Gestation Age				< 0.001
Preterm	22	95.3	4.7	
Term	45	98.1	1.9	
Preeclampsia				0.528
Yes	0	100	0.0	
No	67	97.6	2.4	
Low birth weight				0.009
Yes	13	95.3	4.7	
No	54	97.8	2.2	

### RBF- result based finance

Multivariate analysis was undertaken separately for stillbirths classified as fresh (Table 3) and macerated (Table 4). The odds of both types of stillbirth were increased at RBF relative to non-RBF sites after controlling for variables that were significant in bivariate analyses against RBF. These were preterm birth and low birth weight, resulting in an adjusted odds ratio (aOR) for fresh (intrapartum) losses of 2.67, 95%CI 1.24 to 5.57, *P* = 0.01. Thus, considering gestational age, and low birth weight as possible confounders, RBF status appears to be adverse rather than protective against fresh stillbirth with an aOR of 2.67.

**Table 3:** Fresh stillbirth: adjusted odds of associations with selected variables

Variable	Category’ *Reference group*	aOR	95% CI	*P* Value
RBF	Yes (*n* = 25) *No (n = 10)*	2.67	1.24–5.57	0.010
Gestation age	Preterm (*n* = 8) *Term (n = 27)*	1.61	0.68–3.66	0.29
Low birth weight	Yes (*n* = 6) *No(n-29)*	1.63	0.66–4.20	0.26

**Table 4:** Macerated stillbirth: adjusted odds of associations with selected variables

Variable	Category’ *Reference group*	aOR	95% CI	*P* Value
RBF	Yes (*n* = 27) *No(n = 5)*	7.27	2.74–19.25	< 0.001
Gestation age	Preterm (*n* = 14) *Term(n = 18)*	5.11	2.20–10.07	< 0.001
Low birth weight	Yes (*n* = 7) *No (=25)*	1.58	0.65–3.99	0.312

### Multiple logistic regression model of RBF and other factors associated with macerated stillbirths

The odds of macerated stillbirth were 7-fold higher when a mother delivered at an RBF site (aOR = 7.27) CI: 2.74 to 19.25, *P* < 0.001). An association between gestational age and macerated stillbirth was significant (aOR 5.11 CI: 2.20–10.07, *P* < 0.001). Thus, adjusting for prematurity slightly increased the apparent adverse association with delivery at an RBF site (Table 4) andas for fresh stillbirth risk, RBF status appears adverse and not to be a factor protective against the risk of macerated stillbirth.

## Discussion

We identified 67 stillbirths out of the 2772 births analyzed from the four hospital registers representing 24/1000 live births, a burden of mortality that is closely comparable to the current national estimate [3, 14]. The stillbirth rate identified at RBF sites was 37/1000 live births, substantially higher than at the non-RBF sites which recorded 10/1000 live births.

Intrapartum (fresh) stillbirth is considered to be a measure of the quality of intrapartum care or may reflect inadequacies in antenatal care such that those developing complications are not identified in a timely manner. In our study, stillbirth was significantly more common in RBF sites compared to non-RBF sites: this represents an unintended effect of the financing intervention. In a previous report drawing on experience from Malawi that explored intended and unintended effects of performance-based financing, it was found that apart from a positive impact of PBF on service delivery, unintended effects may occur owing to implementation realities. These include health system factors such as inadequate human resources to cater for the increased service load induced by the intervention [15]. The observed increase in the number of stillbirths at RBF sites could also have resulted from improved documentation and record keeping. This was emphasised in the RBF4MNH program implementation and of course absent at non RBF sites. As an indication that quality of record keeping may indeed partly explain our findings, it was more difficult to locate maternity registers at the non-RBF sites during data collection.

The approximately equal proportions of fresh and macerated stillbirth observed here is consistent with other reports from southern Africa where half or more of the losses were intrapartum [16]. In Gambia, similar findings were attributed to the non-use of the partograph and obstetric complications during intrapartum period [17]. In an evaluation of RBF4MNH impact in Malawi, there was no effect observed on the clinical performance of labor monitoring and partograph documentation with around half of women monitored with complete partographs [8]. This observation is consistent with our findings and may serve to discount the possibility that the apparent adverse effect of the intervention could be entirely a result of improved record keeping.

Previous systematic reviews have found that the most common factors associated with stillbirth in low resource countries were the lack of adequate antenatal care, lack of a skilled attendant at delivery, low socio-economic status, poor nutrition, previous stillbirths and advanced maternal age [18–20]. In the RBF4MNH evaluation study, utilization rates for other maternal care services, specifically timely first antenatal care and at least 4 antenatal care visits, was found to stagnate at very low levels [8]. In Nigeria, over half (57.0%) of the mothers with stillbirths had no antenatal care [21]. Although evidence supporting a direct and linear relationship between antenatal care and stillbirth is lacking, some of the apparent adverse effect of RBF4MNH could also be attributed to low antenatal care utilization as reported in the RBF4MNH final results, even noting the higher overall uptake of antenatal care in Malawi than some other regional countries [8]. Antenatal care can potentially serve as a platform to deliver interventions to improve the quality maternal care and early detection of risk factors that may lead to stillbirths [22]. Several factors beyond those explored in this study could also have contributed to the increase in the number of stillbirths in the RBF4MNH facilities. These factors include increase in service utilization due to incentives given leading to pressure of work, hence affecting quality of care given to the mother during labour and delivery [8].

In this study, a majority of the stillbirths were recorded in the mothers aged between 25 to 34 years with the least being recorded in adolescents below the age of 18. Although age did not show any statistical significance and trends, other subgroups have potentially programmatic significance. For example, a study conducted in Ghana reported that the lifetime risk of stillbirth was 40% higher among adolescents as compared to older mothers [23]. Similarly, a multicountry study conducted to describe the pregnancy outcomes among adolescent mothers reported that a high risk of stillbirth was found among all adolescent age groups, but the risk was significant only among adolescent mothers aged 16–17 years [24]. However, the finding of no association of stillbirths with age observed in this study is likely to reflect the small numbers in this group and should not detract from messages that adolescent reproductive health should remain a priority for both national governments and the global health community.

Preterm birth was significantly associated with stillbirth (33%) and this was observed at both RBF and non-RBF sites. A referral hospital-based study in Malawi also identified preterm birth as a factor associated with stillbirth, and recorded 67% of stillbirths who were preterm (Chilinda GK. Aetiology of stillbirths and adverse newborn outcomes at Queen Elizabeth Central Hospital, Blantyre, Malawi. [Dissertation] Zomba: University of Malawi; 2017 [Unpublished]). The variations in the reported percentages could be due to the difference in study design and the setting, but overall the link between stillbirth risk and preterm birth is an established one [25]. Thus, increasing attention to interventions to prevent preterm birth and stillbirth, alongside increasing investment for the health and wellbeing of mothers, will accelerate progress for these maternal, fetal, and newborn outcomes.

We found no positive impact of RBF on stillbirth despite the interventions targeting the quality care of women both antenatally and during labour. In Mozambique, Pakistan, and India, a large study also showed no impact on stillbirth of a very large community intervention [26]. These programme experiences illustrate how complex a challenge prevention of stillbirth is, as components addressing population-level risk factors and the fine detail of maternity care arrangements across the continuum and especially at the critical time of labour and delivery all need to converge in an effective manner. In some settings the approach has given positive results. In Rwanda where the “pay for performance” intervention, similar in concept to RBF, there was an increase in utilization and quality of maternal and child care services [27]. In Bangladesh, a pay for performance strategy in MNH improved the volume of services provided although it did not address the quality of care [28]. Generally with RBF, improvements have been observed in service utilization but impact on quality has been harder to demonstrate. A recent analysis of the Bangladesh Maternal Health Voucher Scheme indicates a positive impact on access to services especially for marginalized women and on the completeness of antenatal care [29, 30]. Such impact might be expected to feed through to a reduction in stillbirth risk but evidence for this remains elusive to date.

### Study strengths and limitations

The use of cross-sectional data only allows associations to be established, but not causality. However, a strength of the study was the inclusion of a comparison group. The intervention sites and the comparison sites were at the same level in the health system at different locations.

The study results may not represent the whole population of Malawi because only selected district hospitals were considered, and data collection did not include health centres. This study used routinely collected gestation and birth weight data and we were not able to formally confirm clinical causes of stillbirth. We used the traditional clinical classification of stillbirth as ‘fresh’ or ‘macerated’ in order to enable generalizability to the routine service setting where these terms are in use: this is known to be imprecise and there may well have been miss-classification between these categories. However, the main findings would not have been affected by some misclassification.

## Conclusion and recommendations

We were not able to show positive effects of RBF4MNH on stillbirth risk. These findings highlight the need to re-focus interventions onto quality of care and close gaps in program implementation of RBF that might be preventing attainment of potential gains with regard to stillbirth prevention, notably intrapartum care arrangements. We consider that fresh stillbirth is a useful marker for intrapartum care hence an intervention should focus on it as a key indicator. Large scale programmatic investment should be underpinned by rigorous implementation research.

Notwithstanding the limitations of clinical classification without formal perinatal audit, recording of fresh stillbirth and macerated stillbirth in the Health Management Information System (DHIS-2) would enable routine capture of data from the districts for monitoring, planning and implementation of programs targeting care before and during delivery.

## Data Availability

Data will be available from the authors upon request made.

## Abbreviations

COMREC: College of Medicine Research and Ethics Committee
DHIS: District Health Management Information System
DHMT: District Health Management Team
ENAP: Every Newborn Action Plan
FANC: Focused Antenatal Care
FSB: Fresh stillbirth
MDHS: Malawi Demographic and Health Survey
MoH: Ministry of Health
MNH: Maternal and Newborn Health
MSB: Macerated stillbirth
PBF: Performance Based Finance
RHD: Reproductive Health Directorate.
SB: Stillbirth
QECH: Queen Elizabeth Central Hospital
RBF4MNH: Result Based Finance for Maternal and Newborn Health
