# Correction to: Impact of a maternal and newborn health results-based financing intervention (RBF4MNH) on stillbirth: a cross-sectional comparison in four districts in Malawi

**DOI:** 10.1186/s12884-021-03961-9

**Published:** 2021-06-24

**Authors:** Regina Makuluni, William Stones

**Affiliations:** 1 College of Medicine, The University of Malawi, Private Bag 360, Blantyre, Malawi; 2 Center for Reproductive Health, College of Medicine, The University of Malawi, Private Bag 360, Blantyre, Malawi

**Correction to: BMC Pregnancy Childbirth 21, 417 (2021)**

**https://doi.org/10.1186/s12884-021-03867-6**

Following publication of the original article [1], the authors identified an error in the affiliations.

The incorrect affiliations are:

^1^College of Medicine, The University of Malawi, Private Bag 360, Blantyre, Malawi, South Africa

^2^Center for Reproductive health, College of Medicine, The University of Malawi, Private Bag 360, Blantyre, Malawi, South Africa.

The correct affiliations are:

^1^College of Medicine, The University of Malawi, Private Bag 360, Blantyre, Malawi

^2^Center for Reproductive health, College of Medicine, The University of Malawi, Private Bag 360, Blantyre, Malawi

The original article [1] has been corrected.
